# On the Embodiment of Social Cognition Skills: The Inner and Outer Body Processing Differently Contributes to the Affective and Cognitive Theory of Mind

**DOI:** 10.3390/brainsci12111423

**Published:** 2022-10-23

**Authors:** Silvia Canino, Simona Raimo, Maddalena Boccia, Antonella Di Vita, Liana Palermo

**Affiliations:** 1 Department of Health Sciences, Magna Graecia University of Catanzaro, 88100 Catanzaro, Italy; 2 Department of Medical and Surgical Sciences, Magna Graecia University of Catanzaro, 88100 Catanzaro, Italy; 3 Department of Psychology, Sapienza University of Rome, 00185 Rome, Italy; 4 IRCCS Santa Lucia Foundation, 00179 Rome, Italy; 5 Department of Human Neuroscience, Sapienza University of Rome, 00185 Rome, Italy

**Keywords:** social cognition, theory of mind, embodiment, embodied cognition, interoception, interoceptive sensibility, bodily sensations, body image, body representation, body schema

## Abstract

A specific interpretation of embodiment assigns a central role to the body representations (BR) in cognition. In the social cognition domain, BR could be pivotal in representing others’ actions and states. However, empirical evidence on the relationship between different BR and social cognition, in terms of Theory of Mind (ToM), in the same sample of participants is missing. Here, this relationship was explored considering individual differences in the action-oriented BR (aBR), nonaction-oriented BR (NaBR), and subjective predisposition toward internal bodily sensations (interoceptive sensibility, ISe). Eighty-two healthy adults were given behavioral measures probing aBR, NaBR, ISe, and affective/cognitive ToM. The results suggest that NaBR, which mainly relies on exteroceptive signals, predicts individual differences in cognitive ToM, possibly because it can allow differentiating between the self and others. Instead, the negative association between affective ToM and ISe suggests that an alteration of the internal body state representation (i.e., over-reporting interoceptive sensations) can affect emotional processing in social contexts. The finding that distinct aspects of the body processing from within (ISe) and from the outside (NaBR) differently contribute to ToM provides empirical support to the BR role in social cognition and can be relevant for developing interventions in clinical settings.

## 1. Introduction

Theory of Mind (ToM), together with imitation and empathy, is a core component of social cognition that affects how we interact with others [1,2]. Specifically, ToM has been defined as “the ability to represent one’s own mental states and those of others” [2]. Despite the growing interest in this construct, it is critical to emphasize that there is still some terminological confusion in the literature, and, for example, the terms ToM and perspective-taking are used interchangeably by different authors (see for such an account [2,3]).

The inferred mental states are not only cognitive but also concern emotions and feelings. Indeed, current evidence has shown that ToM is a multidimensional construct that can be dissociated into two components: (i) *affective ToM*, defined as the ability to infer the emotions or feelings of another individual; (ii) *cognitive ToM*, defined as the ability to infer the thoughts, beliefs, or intentions of another individual [4,5]. 

Neuroimaging data also support the view that cognitive and affective ToM are distinct processes. Indeed, although both types of ToM are associated with brain regions such as the temporoparietal junction and precuneus, functional imaging studies in healthy adults and structural imaging studies in patients with dementia suggest that cognitive ToM uniquely engages the dorsomedial prefrontal cortex and dorsolateral prefrontal cortex, while affective ToM uniquely engages the amygdala, basal ganglia, ventromedial prefrontal cortex, and inferior frontal gyrus [4]. Similarly, lesion studies in individuals with unilateral brain damage show that deficits in cognitive and affective ToM relate to dissociated lesion patterns, respectively, in the prefrontal and insular cortex [6].

An outstanding open question in ToM research concerns what determines individual differences in inferring others’ cognitive and affective mental states. In this vein, some studies have focused on other cognitive skills, such as executive functions [7,8]. However, several empirical findings have shown the presence of interesting interactions between high-level cognitive functions, including social cognition, and the sensorimotor and visceral systems, indicating that we use our own bodily processes to understand our own and others’ emotional experience [9,10,11]. Additionally, several findings suggest that the understanding of others’ states and feelings activates the same brain areas that are active when the observer experiences that state or feeling [12,13,14]. According to these “embodied” theories, much of the cognitive processes are mediated by the body’s control systems [1,15]; thus, individual differences in bodily processing could account for individual differences in cognition, including ToM. 

Although some embodiment theories underline the role of the body in cognition, mainly referring to the body’s anatomy or activities, an alternative interpretation assigns a central role to the body representations [16]. Indeed, following Goldman and de Vignemont [16], one of the most promising interpretations of the notion of embodiment, with references to the social cognition domain, considers *the mental representations of the body* causal for cognition, emphasizing that mental states and processes are “embodied” because of their various bodily formats (e.g., motoric, somatosensory, and interoceptive formats) or contents. Thus, although initially the embodiment theoretical perspective only emphasized a relationship between the material body and cognitive abilities, today, the influence of the body in terms of bodily mental representations, including sensorimotor and visceral systems, is increasingly clear (see also [17]). However, whether individual differences in body representations have a role in social cognition components, including ToM, is still being debated at the empirical level.

### 1.1. Higher-Order Body Representations and Social Cognition

Here, we will refer to higher-order, more cognitive representations of the body that go beyond the somatosensory homunculus [18]. According to their functional role, higher-order body representations (BR) may be classified into action-oriented BR (aBR) and nonaction-oriented BR (NaBR) [19,20,21,22]. The aBR, or body schema, corresponds to a dynamic sensorimotor representation of the body that guides actions and movements. The NaBR includes all the other perceptual, conceptual, or emotional representations of the body that are not used for action.

There are some suggestions for a possible relationship between the body schema and ToM. For example, Gunia and colleagues [23] have recently summarized findings suggesting that visuospatial perspective-taking, a process strictly related to ToM (see [24]), engages brain areas coding for the body schema, implying a possible relationship between these constructs. The possible relationship between ToM and the body schema is also evident through the second reading of behavioral studies that have used mental rotation tasks with bodily stimuli (i.e., tasks that are classically used as measures to evaluate the body schema) in healthy adults [25] and children [26].

An efficient NaBR, in terms of an efficient topological map of the body that contains information about the borders of the body, the location of body parts, and distance relations between body parts (i.e., the structural representation of the body, see [27,28]), could be particularly critical for the ability to differentiate between the self and the other that, in turn, is considered pivotal to correctly attribute mental and affective states to their origin [29,30].

The contribution of the NaBR to social cognition skills can be inferred by studies that have investigated the BR alteration in terms of body ownership, using experimentally induced illusions, such as the rubber hand illusion. Indeed, following the neurocognitive model of body ownership by Tsakiris [31], a critical process during the rubber hand illusion is the comparison between the pre-existing BR that contains the visual and structural properties of the body (i.e., a NaBR) and the rubber hand. For example, a positive correlation between stronger “proprioceptive drift” (i.e., a localization bias of the actual hand towards the artificial hand) and higher levels of empathy has been described both in healthy individuals (e.g., [32]) and clinical samples (e.g., [33]).

A relationship between NaBR and ToM can also be inferred by referring to the literature on autism and schizophrenia. Indeed, in these clinical conditions, NaBR and ToM deficits have been reported, and a recent model proposed by Tordjman and colleagues [34] suggests that bodily self-consciousness disorders in schizophrenia and autism would result in problems of self–other differentiation, leading to impaired social cognition, including deficits of ToM and empathy.

However, more direct empirical evidence on the relationship between individual differences in both aBR and NaBR on the one hand and ToM on the other in the same sample of participants is missing. 

### 1.2. Interoception and Social Cognition

Social cognition research is progressively targeting interoceptive processing as an important source of individual differences in social abilities.

The definition of interoception has evolved over the years. Initially, this concept overlapped with that of visceral perception but, more recently, has been defined as the sense of the physiological condition of the whole body [35,36]. It thus refers to the processing of a variety of internal bodily signals, spanning from the heartbeat to the itch and “air hunger” [35].

Interoception can be operationalized along three main dimensions: (i) *interoceptive accuracy* (IAcc), namely, the performance on objective tasks, such as heartbeat detection tasks; (ii) *interoceptive sensibility* (ISe), the self-perceived tendency to focus on interoceptive signals, tested using questionnaires; (iii) *interoceptive awareness* (IAw), the metacognitive awareness of interoceptive accuracy (for an overview, see [37]).

Some studies suggest that IAcc can be used as a predictor of the representation of one’s own body and the boundaries between oneself and the other, playing, therefore, a pivotal role in social cognition [38,39]. In particular, Tajadura-Jiménez and Tsakiris [39] found that individuals with lower IAcc showed larger malleability of the self-other boundaries, as evaluated using the enfacement illusion. 

However, studies about the role of individual differences in the various interoceptive dimensions on a core component of social cognition, such as empathy, show mixed results. Indeed, better IAcc and ISe have been associated with higher empathy, in terms of a higher tendency to share and understand other emotions and feelings (for IAcc, see [40,41]; for Ise, see [42,43]) but some studies have failed to report such an association (see [39,44]).

To the best of our knowledge, only one study by Shah and colleagues [45] has investigated the role of individual IAcc differences in affective and cognitive ToM. Specifically, in this study, 72 participants were given an IAcc task, that is, the Heartbeat Tracking task [46], and a measure of ToM, that is, the Movie for the Assessment of Social Cognition (MASC; [47]). Interestingly, there was only a significant association between IAcc and the performance on items requiring the representation of another’s emotion. In contrast, no association was found when the representation of emotional states was not required, suggesting that interoception can help us accurately represent mental states in situations where “the process is reliant on emotional or, otherwise, interoceptive information” [45]. 

Instead, to the best of our knowledge, no studies have investigated the role of individual differences in the ISe dimension in ToM (for an overview, see also [3]).

### 1.3. The Present Study

The purpose of the present study is to bring together these aspects of bodily processing, which have previously been independently and indirectly studied, to explore the relationship between cognitive and affective ToM on the one hand and ISe, aBR, and NaBR on the other in the same sample of healthy adults, using an individual differences approach. Additionally, the performance in control tasks will be considered to regress out the contribution of cognitive skills not related to body processing.

We predict that individuals with better BR will report higher performance in the *cognitive* component of ToM based on the idea that they can better co-represent themselves and others and, thus, correctly attribute mental states to their origin.

Considering the previous literature about IAcc (see [3,45]), we expect to find a relationship only between ISe and the *affective* component of ToM.

## 2. Materials and Methods

### 2.1. Participants

To determine the minimum sample size necessary for the study, a priori power analysis was performed (assuming a power of 0.8; *α* = 0.05, *r* = 0.31) using the software G∗Power 3.1.9.7, freely available at https://www.psychologie.hhu.de/arbeitsgruppen/allgemeine-psychologie-und-arbeitspsychologie/gpower.html (accessed on 20 October 2021) [48]. The analysis revealed that a sample size of at least 79 participants would be necessary. The estimation of a correlation of 0.31 was based on a previous study on interoception and theory of mind [45]. Eighty-two healthy individuals (sixty-one women, mean age = 28.6 years, SD = 12.4; twenty-one men, mean age = 29.9 years, SD = 9.3) participated in the study.

All participants had normal or corrected to normal vision and no history of neurological or psychiatric conditions. Before taking part in the study, all participants gave informed consent. Approval was obtained from the local ethics committee of the Calabria Region Ethical Committee, Catanzaro, Italy (protocol number 400, 18 November 2021) in accordance with the criteria laid down in the 1964 Declaration of Helsinki.

### 2.2. Behavioral Testing

#### 2.2.1. Procedure

Participants were asked to complete a self-administered online battery of tasks and questionnaires presented in a randomized order, using the experiment builder “Testable”. Written instructions were provided on the screen before performing each task/questionnaire. Since the precise recording of response times is difficult in online experiments, we only focused on answer accuracy.

#### 2.2.2. Assessment of the Interoceptive Sensibility

To assess ISe, participants completed the Self-Awareness Questionnaire (SAQ; [49]). The SAQ is a self-report questionnaire composed of 35 items developed specifically to evaluate the frequency of common body feelings. Items are clustered into two domains, one related to visceral feelings (e.g., “I feel my heart thudding”) and the other to somatosensory feelings (e.g., “I feel my palms sweaty”). The participants were asked to read each item carefully and to rate it by selecting one response option (i.e., never; sometimes; often; very often; always). Each item is scored on a five-point Likert scale (0 = never; 1 = sometimes; 2 = often; 3 = very often; 4 = always), and the total score is given by the sum of the responses of all items providing a score range of 0 to 140. Higher scores indicate higher levels of ISe.

#### 2.2.3. Assessment of Body Representations

According to a functional distinction of the higher-order BR into aBR (i.e., body schema) and NaBR (i.e., body structural representation or visuospatial body map) [20,28], the BR assessment was performed using a specific battery that included aBR and NaBR tasks.

The aBR was evaluated using a Hand Laterality Task (HLT; adapted from [28,50]. In this task, the participants were asked to decide on the laterality of a single hand drawing (48 stimuli, 24 left and 24 right hands), which was presented at different degrees of rotation angle (i.e., 0, 45, 90, 135, 180, 225, 270, 315 degrees). The participants had to decide whether the rotated hand displayed in the center of the screen was a left or a right hand by mentally rotating it and selecting one of the two response options (i.e., a left and a right hand not rotated) shown on the left and right bottom part of the computer screen. The stimuli included 16 drawings of the right and left hand displayed in a back view. The task included 48 trials, and the response accuracy was recorded for each trial. Individual scores ranged from 0 to 48, with higher scores indicating better performance.

The NaBR was evaluated using a modified version of the Frontal Body Evocation task (FBE) [28,51]. First, a picture of a human body was displayed on the screen for 10 s. Then, the participants were asked to decide if a specific body part (i.e., left/right hand, left/right arm, left/right leg, left/right foot) was correctly or incorrectly positioned, having only the head or the waist as a reference. The task included 48 trials, and response accuracy was recorded for each trial. Individual scores ranged from 0 to 48, with higher scores indicating better performance.

To disentangle the BR contribution from general cognitive abilities necessary to perform BR tasks (e.g., mental imagery, visuospatial skills, attention, working memory, decision making), the participants were also given two control tasks similar to tasks probing BR for features such as presentation and response modalities but not including body stimuli.

The control task for the aBR task was the Object Laterality Task (adapted from [28]). The participants were asked to decide on the laterality of a non-body stimulus (i.e., a flower), which was presented on the screen at different degrees of rotation angles (i.e., 0, 45, 90, 135, 180, 225, 270, 315 degrees). The participants had to decide whether the rotated flower was with a leaf positioned at the right or at the left base of the stem by mentally rotating it and selecting one of the two response items (i.e., two not-rotated flowers, one flower with a leaf positioned at the left of the stem, and one flower with a leaf positioned at the right of the stem) shown in the bottom-left and -right parts of the computer screen. The task included 48 trials, and the response accuracy was recorded for each trial. Individual scores ranged from 0 to 48, with higher scores indicating better performance.

Finally, the control task for the NaBR task was the Christmas Tree Task (adapted from [28]). First, a picture of a Christmas tree was displayed on the screen for 10 s. Then, the participants were asked to decide if a specific part of the tree (i.e., the left/right upper branches, left/right mid-upper branches, left/right mid-lower branches, left/right lower branches with the trunk) was correctly or incorrectly positioned, having only the star tree topper or the jar as a reference. The task included 48 trials, and the response accuracy was recorded for each trial. Individual scores ranged from 0 to 48, with higher scores indicating better performance.

#### 2.2.4. Assessment of Affective and Cognitive ToM

ToM was evaluated by means of two tasks that have been used in previous studies to differentiate between affective and cognitive ToM (see, for example, [52,53,54]).

The Emotion Attribution Task (EAT; [55]), which investigates the ability to attribute *emotional* states to others, was used to evaluate the affective component of the ToM. The EAT consists of 35 short stories describing emotional situations. Five stories describe scenes of sadness, five of fear, five of embarrassment, five of disgust, five of happiness, five of anger, and five of envy.

For each story, the participants were asked to report what the main protagonists felt in that situation. The total score ranges from 0 (worst performance) to 35 (best performance).

Moreover, we subdivided the various stories into two domains: stories eliciting negative emotion attributions (negEAT) and stories eliciting positive emotion attributions (posEAT); for each domain, we calculated the percentage of correct responses.

The Advanced Test of ToM (ATT; [56]; Italian version [57]), which investigates the ability to attribute *mental* states to others, was used to evaluate the cognitive component of the ToM. The ATT consists of 13 stories describing several situations in which two or more characters interact in social contexts. The participants were asked to explain why the characters behaved as they did. The total score ranges from 0 (worst performance) to 13 (best performance).

### 2.3. Statistical Analyses

To verify the normality of data distribution, we used the Kolmogorov–Smirnov test. Due to the non-normal distribution of the experimental variables, non-parametric analyses were performed.

To investigate the association between the affective and cognitive ToM and BR, partial Spearman correlations were performed between the ToM tasks’ (i.e., negEAT, posEAT, and AAT) and the BR tasks’ (i.e., HLT and FBE) scores, regressing out the contribution of the performance in the control tasks (i.e., the performance in the Object Laterality Task for the HLT and the performance in the Christmas Tree Task for the FBE).

To investigate the association between ISe and the affective and cognitive components of ToM, Spearman correlation coefficients between the ToM tasks (i.e., negEAT, posEAT, and AAT) and the SAQ total score were calculated.

In addition, to further explore the relation between ToM and BR/ISe, stepwise linear regressions on rank-transformed variables [58] were run to identify the possible predictors of the cognitive (i.e., ATT) and affective ToM (i.e., negEAT, posEAT) out of the following variables: SAQ, HTL, and FBE. Considering the lack of significant correlations between the cognitive and affective ToM and the control tasks (i.e., Object Laterality Task and HLT; see Supplementary Materials), the control tasks were not entered in the stepwise linear regressions. However, for the ToM tasks in which a significant contribution of the BR tasks was identified, additional regression analyses, using as predictors both the rank-transformed BR and relative control task, were performed to further rule out a possible contribution of the control tasks (see Supplementary Materials).

## 3. Results

Descriptive statistics for ISe, aBR, NaBR, and ToM tasks are reported in Table 1.

Significant correlations were found between aBR/NaBR and the cognitive component of ToM even when the contribution of control tasks was taken into account. In particular, positive partial correlations emerged between AAT and FBE (*r_rho_* = 0.27, *p* < 0.01), and between AAT and HLT (*r_rho_* = 0.21, *p* = 0.03). In contrast, no significant associations were found between aBR/NaBR and the affective component of ToM (see Table 2). In addition, no significant correlations were found between the control tasks and the cognitive/affective ToM (see Table S1 in Supplementary Materials).

Significant correlations were also found between SAQ scores and the affective component of ToM. In particular, the SAQ negatively correlates with the attribution of negative emotions (negEAT; *r_rho_* = −0.22, *p* = 0.04). In other words, individuals with a higher level of ISe had more difficulty in the correct attribution of negative emotions. No significant association was found between the SAQ and the cognitive component of ToM (see Table 2).

The stepwise linear regression analyses (see Table 3) showed that the affective ToM, in terms of attribution of negative emotions (i.e., negEAT), was negatively predicted by the SAQ total score (*Beta* = −0.22, *t_1,80_* = −2.05, *p* = 0.04; *R^2^* = 0.05), while none of the variables predicted the affective ToM in terms of attribution of positive emotions (i.e., posEAT). Concerning the cognitive ToM, the analysis showed that the AAT scores were significantly predicted by the FBE scores (*Beta* = 0.26, *t_1,80_* = 2.43, *p* = 0.02; *R^2^* = 0.07). In addition, a regression analysis including as predictors the FBE and the respective control task (i.e., Christmas Tree Task) showed an exclusive contribution of the FBE scores to the AAT performance (see Table S2 in Supplementary Materials).

## 4. Discussion

In this study, following the hypothesis that mental representations with bodily contents and in various bodily formats could play a pivotal role in social cognition [16], we investigated whether and to what extent individual differences in aBR, NaBR, and ISe predicted individual differences in cognitive and affective ToM in healthy adults.

Based on the hypothesis that individuals with more efficient BR can better co-represent themselves and others, we predicted that better aBR and NaBR would result in better performance in a task probing the *cognitive* component of ToM. Furthermore, considering previous reports of the better performance of individuals with higher accuracy in detecting their heartbeat (i.e., higher IAcc) only in ToM tasks requiring the representation of another’s emotional states (see [45]), we expected to find a relationship only between ISe and the *affective* component of ToM.

Concerning higher-order BR, consistently with our predictions, we observed a significant association between the cognitive ToM on the one hand and both aBR and NaBR on the other, also when the performance in control tasks non-involving body processing was taken into account. Using control tasks without body stimuli and similar to the BR ones for all the other features is a strength of the study since individual differences in cognitive abilities involved in our tasks, such as attention, working memory, and visuospatial skills could contribute to performance in ToM tasks (see, for example, [59,60]). However, since sex differences in ToM skills have been reported [61,62,63], caution should be taken in drawing definitive conclusions since our sample included mainly women.

Our finding ties in well and expands on previous studies that suggested a link between BR and social cognition skills (e.g., [23,25,32]), providing more direct empirical evidence about the relationship between cognitive ToM and individual differences in aBR and NaBR in the same sample of healthy adults. Notably, our regression analysis results showed that individual differences principally in NaBR performance predict the capability to represent the cognitive mental state of other individuals. The efficient processing of visuospatial relations and distances among body parts (i.e., NaBR) could be particularly critical in cognitive ToM because it can allow differentiating between the self and the other, a skill considered pivotal to correctly attribute mental states to their origin [29]. Indeed, although a shared representation network underpins our ability to represent our own mental states and that of others, there is not a complete overlap that would lead to confusion, and successful social interactions also imply distinguishing the representations of the self from that of others [29]. We argue that an efficient NaBR could be particularly relevant for navigating within these shared representations.

As expected, we also observed an association between the ISe and the affective ToM when evaluated considering the attribution of negative emotions. Indeed, a higher level of ISe predicted a lower performance in understating the emotion felt by the main protagonist of a story in a specific situation. Thus, our results suggest that heightened ISe can sometimes occur at the expense of the correct processing of emotional information in social contexts.

The negative association between ISe and affective ToM is consistent with the evidence that the more frequent monitoring of one’s internal body states is associated with self-reported difficulties in the ability to perceive, express, and recognize emotions typical of alexithymia [43,64]. Indeed, people with alexithymia tend to over-report physical symptoms [65] and show difficulties in recognizing emotions expressed by others (e.g., [66,67]). This evidence is further supported by a recent study that suggests that individuals with alexithymia perceive their visceral sensations more intensively, reporting a higher level of ISe, without being able to interpret them cognitively [68]; this, in turn, would prevent selecting adequate strategies for regulating emotions [67,68].

Considering the positive association between IAcc and affective ToM reported in the study by Shah and colleagues [45], our finding of a negative association between the ability to attribute negative emotion and ISe further points to the idea that ISe and IAcc are two distinct constructs [31,69] that can impact social abilities differently. Furthermore, in a recent study by von Moher and colleagues [30], individuals with higher IAcc displayed lower emotional egocentricity bias (i.e., a lower tendency to use their own emotional state when judging the emotional experience of other individuals) when compared with individuals with lower IAcc at diastole/baseline, consistently with the study by Shah and colleagues [45]. However, when the other’s emotional state was presented at the point of maximum interoceptive impact (i.e., at systole), the opposite pattern was observed, that is, an increased emotional egocentricity bias in individuals with higher IAcc, suggesting a more complex picture of the relationship between IAcc and affective ToM.

The fact that the individual differences in the level of ISe predicted the ability to attribute only negative emotions deserves further comment. In particular, this association is consistent with the finding that bodily signals influence the flexible allocation of attentional resources to threat signals [70] and with the idea that the link between interoception and affective ToM could be stronger for specific emotional states (e.g., fear, pain, and sadness; for such an account, see also [3]).

Overall, these results underline that the body processing from within and from the outside specifically and differently contributes to the two components of ToM, supporting the idea that mental representations with bodily contents or mental representations in bodily format may play a causal role in social cognition [10].

## 5. Conclusions

In conclusion, the current findings suggest that a body representation that mainly relies on exteroceptive signals (i.e., NaBR), that is, signals from the outside of the body (e.g., vision), specifically predicts individual differences in understanding other mental states (cognitive ToM), possibly because it can allow differentiating between the self and the other. On the other hand, an alteration of the internal body state representation, as defined in terms of over-reporting, on a questionnaire, the experience of interoceptive sensations, can affect the attribution of negative emotions.

The present results can have theoretical and clinical relevance. Theoretically, they expand on previous findings on other social cognition skills [37], providing empirical support to an exciting interpretation of the embodiment that assigns a pivotal role to BR in social cognition [10].

Clinically, these findings can provide new insights for developing innovative interventions. Indeed, considering the presence of BR disorders, interoceptive alterations, and ToM difficulties in pathologies such as schizophrenia and autism [28,71], this knowledge can be valuable in clinical settings to implement interventions aimed at improving the visuospatial processing of the body and at reducing the over-reporting of interoceptive sensations, that, in turn, can enhance ToM skills.

## 6. Limitations and Future Directions

Despite these new insights, some limits and opportunities for future research studies should be acknowledged. First, we focused only on the ISe dimension, using an instrument (i.e., the SAQ) that mainly probes uncomfortable visceral and somatic sensations (e.g., “I feel a burning sensation in my stomach”) [for such account see, [72]). Secondly, our participants were mainly women. Thirdly, the role of other individual differences, such as personality traits, in the ToM abilities was not taken into account. Finally, considering the online nature of the study, we did not focus on reaction times, but this parameter could be more sensitive than the accuracy of an answer to detect individual differences in the task probing aBR (i.e., Hand Laterality Task). Additionally, it should be acknowledged that a possible ceiling effect on the accuracy of the answer in the Hand Laterality Task can affect the correct interpretation of our findings.

Thus, starting from these findings, future lab-based studies should enroll samples better balanced for sex, should use RTs, and consider the processing of comfortable, neutral, and uncomfortable bodily sensations, as well as the role of different interoceptive dimensions (i.e., IAcc, ISe, and IAw) and submodalities (see, for example, [73]). Additionally, future studies should address the possible mediation/moderator role of the body processing in the relation between other kinds of individual differences (e.g., personality traits) and ToM.

## Figures and Tables

**Table 1:** Descriptive statistics for questionnaires and tasks used in the study.

	ISe	aBR	NaBR	Affective ToM (% Correct)		Cognitive ToM
	SAQ	HLT	FBE	negEAT	posEAT	ATT
Mean (SD)	27.4 (11.9)	45.3 (5.78)	35.8 (6.06)	70.2 (12.5)	80.0 (19.1)	8.38 (2.03)
Min–Max	5–54	24–48	20–48	33.3–93.3	20–100	4–13

The table shows the means, SDs, and minimum/maximum scores for the measures probing ISe, aBR, NaBR, and ToM. Note: ISe, interoceptive sensibility; SAQ, Self-Awareness Questionnaire; aBR, Action-oriented Body Representation; HLT, Hand Laterality Task; NaBR, Nonaction-oriented Body Representation; FBE, Frontal-Body Evocation Task; ToM, Theory of Mind; negEAT, negative Emotion Attributions Task; posEAT, positive Emotion Attributions Task; AAT, Advanced Test of ToM.

**Table 2:** Spearman correlation coefficients between the cognitive and affective components of ToM measures on the one hand and the ISe, aBR, and NaBR measures on the other.

			ISe	aBR	NaBR
			SAQ	HLT *	FBE #
**Affective ToM**	negEAT	*r_rho_* *p*	**−0.22** 0.04	−0.02 0.85	0.16 0.16
	posEAT	*r_rho_* *p*	−0.05 0.69	0.02 0.86	0.09 0.41
**Cognitive ToM**	ATT	*r_rho_* *p*	−0.04 0.72	**0.21** 0.03	**0.27** <0.01

The table shows Spearman correlations between the cognitive (ATT) and affective (negEAT; posEAT) ToM tasks on the one hand and the ISe, aBR, and NaBR measures on the other. For the HLT and FBE tasks, the performance in the control tasks was statistically partialed out. Note: * Partial Spearman correlations, controlling for the performance in the Object Laterality Task; # Partial Spearman correlations, controlling for the performance in the Christmas Tree Task. Significant correlations are in bold. ISe, interoceptive sensibility; SAQ, Self-Awareness Questionnaire; aBR, Action-oriented Body Representation; HLT, Hand Laterality Task; NaBR, Nonaction-oriented Body Representation; FBE, Frontal-Body Evocation Task; ToM, Theory of Mind; negEAT, negative Emotion Attributions Task; posEAT, positive Emotion Attribution Task; ATT, Advanced Test of ToM.

**Table 3:** Standardized regression coefficients predicting affective and cognitive ToM.

	Predictor	*Beta*	*t*	*p*
**Affective ToM**				
negEAT	SAQ	−0.22	−2.05	0.04
	*Excluded variables*			
	HLT	0.07	0.68	0.50
	FBE	0.17	1.54	0.13
**Cognitive ToM**				
ATT	FBE	0.26	2.43	0.02
	*Excluded variables*			
	HLT	0.17	1.48	0.14
	SAQ	−0.06	−0.57	0.57

The table shows the results of the stepwise linear regressions to identify the possible predictors of the cognitive (ATT) and affective ToM (negEAT). In particular, the standardized regression coefficients for the variables predicting the performance in the affective and cognitive ToM tasks and for the excluded variables are reported. Note: ToM, Theory of Mind; negEAT, negative Emotion Attributions Task; ATT, Advanced Test of ToM; SAQ, Self-Awareness Questionnaire; HLT, Hand Laterality Task; FBE, Frontal-Body Evocation Task.

## Data Availability

Data are available on reasonable request from the corresponding authors.

## Supplementary Materials

The following supporting information can be downloaded at: https://www.mdpi.com/article/10.3390/brainsci12111423/s1. Table S1. Spearman correlation coefficients between the cognitive and affective components of ToM measures on the one hand and control tasks on the other. Table S2. Results of a multiple regression analysis to explore the effect of the FBE and Christmas Tree Task on the Advanced Test of ToM (ATT) performance.

## References

[1] Gao Q., Ping X., Chen W. (2019). Body Influences on Social Cognition through Interoception. Front. Psychol..

[2] Happé F., Cook J.L., Bird G. (2017). The Structure of Social Cognition: In(Ter)Dependence of Sociocognitive Processes. Annu. Rev. Psychol..

[3] Baiano C., Job X., Santangelo G., Auvray M., Kirsch L.P. (2021). Interactions between Interoception and Perspective-Taking: Current State of Research and Future Directions. Neurosci. Biobehav. Rev..

[4] Healey M.L., Grossman M. (2018). Cognitive and Affective Perspective-Taking: Evidence for Shared and Dissociable Anatomical Substrates. Front. Neurol..

[5] Shamay-Tsoory S.G., Aharon-Peretz J. (2007). Dissociable Prefrontal Networks for Cognitive and Affective Theory of Mind: A Lesion Study. Neuropsychologia.

[6] Corradi-Dell’Acqua C., Ronchi R., Thomasson M., Bernati T., Saj A., Vuilleumier P. (2020). Deficits in Cognitive and Affective Theory of Mind Relate to Dissociated Lesion Patterns in Prefrontal and Insular Cortex. Cortex.

[7] Di Tella M., Ardito R.B., Dutto F., Adenzato M. (2020). On the (Lack of) Association between Theory of Mind and Executive Functions: A Study in a Non-Clinical Adult Sample. Sci. Rep..

[8] Wade M., Prime H., Jenkins J.M., Yeates K.O., Williams T., Lee K. (2018). On the Relation between Theory of Mind and Executive Functioning: A Developmental Cognitive Neuroscience Perspective. Psychon. Bull. Rev..

[9] Damasio A. (2000). The Feeling of Things: Body and Emotion in the Making of Consciousness.

[10] Havas D.A., Glenberg A.M., Gutowski K.A., Lucarelli M.J., Davidson R.J. (2010). Cosmetic Use of Botulinum Toxin-A Affects Processing of Emotional Language. Psychol. Sci..

[11] Wollmer M.A., de Boer C., Kalak N., Beck J., Götz T., Schmidt T., Hodzic M., Bayer U., Kollmann T., Kollewe K. (2012). Facing Depression with Botulinum Toxin: A Randomized Controlled Trial. J. Psychiatr. Res..

[12] Carr L., Iacoboni M., Dubeau M.-C., Mazziotta J.C., Lenzi G.L. (2003). Neural Mechanisms of Empathy in Humans: A Relay from Neural Systems for Imitation to Limbic Areas. Proc. Natl. Acad. Sci. USA.

[13] Gallese V., Goldman A. (1998). Mirror Neurons and the Simulation Theory of Mind-Reading. Trends Cogn. Sci..

[14] Gallese V. (2003). The Roots of Empathy: The Shared Manifold Hypothesis and the Neural Basis of Intersubjectivity. Psychopathology.

[15] Dijkerman C., Lenggenhager B. (2018). The Body and Cognition: The Relation between Body Representations and Higher Level Cognitive and Social Processes. Cortex.

[16] Goldman A., de Vignemont F. (2009). Is Social Cognition Embodied?. Trends Cogn. Sci..

[17] Raimo S., Martini M., Guariglia C., Santangelo G., Trojano L., Palermo L. (2022). Editorial: Body Representation and Interoceptive Awareness: Cognitive, Affective, and Social Implications. Front. Psychol..

[18] Haggard P., Wolpert D.M., Freund H.J., Jeannerod M., Hallett M., Leiguarda R. (2005). Disorders of Body Scheme. Higher-Order Motor Disorders.

[19] De Vignemont F. (2011). Embodiment, Ownership and Disownership. Conscious. Cogn..

[20] Di Vita A., Boccia M., Palermo L., Guariglia C. (2016). To Move or Not to Move, That Is the Question! Body Schema and Non-Action Oriented Body Representations: An FMRI Meta-Analytic Study. Neurosci. Biobehav. Rev..

[21] Pitron V., de Vignemont F. (2017). Beyond Differences between the Body Schema and the Body Image: Insights from Body Hallucinations. Conscious. Cogn..

[22] Boccia M., Di Vita A., Palermo L., Nemmi F., Traballesi M., Brunelli S., De Giorgi R., Galati G., Guariglia C. (2020). Neural Modifications in Lower Limb Amputation: An FMRI Study on Action and Non-Action Oriented Body Representations. Brain Imaging Behav..

[23] Gunia A., Moraresku S., Vlček K. (2021). Brain Mechanisms of Visuospatial Perspective-Taking in Relation to Object Mental Rotation and the Theory of Mind. Behav. Brain Res..

[24] Perner J., Roessler J. (2012). From Infants’ to Children’s Appreciation of Belief. Trends Cogn. Sci..

[25] Xie J., Cheung H., Shen M., Wang R. (2018). Mental Rotation in False Belief Understanding. Cogn. Sci..

[26] Lehmann J., Jansen P. (2019). The Relationship between Theory of Mind and Mental Rotation Ability in Preschool-Aged Children. Cogent Psychol..

[27] Schwoebel J., Coslett H.B. (2005). Evidence for Multiple, Distinct Representations of the Human Body. J. Cogn. Neurosci..

[28] Raimo S., Iona T., Di Vita A., Boccia M., Buratin S., Ruggeri F., Iosa M., Guariglia C., Grossi D., Palermo L. (2021). The Development of Body Representations in School-Aged Children. Appl. Neuropsychol. Child.

[29] Decety J., Sommerville J.A. (2003). Shared Representations between Self and Other: A Social Cognitive Neuroscience View. Trends Cogn. Sci..

[30] Von Mohr M., Finotti G., Villani V., Tsakiris M. (2021). Taking the Pulse of Social Cognition: Cardiac Afferent Activity and Interoceptive Accuracy Modulate Emotional Egocentricity Bias. Cortex.

[31] Tsakiris M. (2010). My Body in the Brain: A Neurocognitive Model of Body-Ownership. Neuropsychologia.

[32] Asai T., Mao Z., Sugimori E., Tanno Y. (2011). Rubber Hand Illusion, Empathy, and Schizotypal Experiences in Terms of Self-Other Representations. Conscious. Cogn..

[33] Cascio C.J., Foss-Feig J.H., Burnette C.P., Heacock J.L., Cosby A.A. (2012). The Rubber Hand Illusion in Children with Autism Spectrum Disorders: Delayed Influence of Combined Tactile and Visual Input on Proprioception. Autism.

[34] Tordjman S., Celume M., Denis L., Motillon T., Keromnes G. (2019). Reframing Schizophrenia and Autism as Bodily Self-Consciousness Disorders Leading to a Deficit of Theory of Mind and Empathy with Social Communication Impairments. Neurosci. Biobehav. Rev..

[35] Craig A.D. (2002). How Do You Feel? Interoception: The Sense of the Physiological Condition of the Body. Nat. Rev. Neurosci..

[36] Garfinkel S.N., Critchley H.D. (2013). Interoception, Emotion and Brain: New Insights Link Internal Physiology to Social Behaviour. Commentary on: “Anterior Insular Cortex Mediates Bodily Sensibility and Social Anxiety” by Terasawa et al. (2012). Soc. Cogn. Affect. Neurosci..

[37] Garfinkel S.N., Seth A.K., Barrett A.B., Suzuki K., Critchley H.D. (2015). Knowing Your Own Heart: Distinguishing Interoceptive Accuracy from Interoceptive Awareness. Biol. Psychol..

[38] Tsakiris M., Tajadura-Jiménez A., Costantini M. (2011). Just a Heartbeat Away from One’s Body: Interoceptive Sensitivity Predicts Malleability of Body-Representations. Proc. R. Soc. B.

[39] Tajadura-Jiménez A., Tsakiris M. (2014). Balancing the “Inner” and the “Outer” Self: Interoceptive Sensitivity Modulates Self–Other Boundaries. J. Exp. Psychol. Gen..

[40] Fukushima H., Terasawa Y., Umeda S. (2011). Association between Interoception and Empathy: Evidence from Heartbeat-Evoked Brain Potential. Int. J. Psychophysiol..

[41] Grynberg D., Pollatos O. (2015). Perceiving One’s Body Shapes Empathy. Physiol. Behav..

[42] Mul C., Stagg S.D., Herbelin B., Aspell J.E. (2018). The Feeling of Me Feeling for You: Interoception, Alexithymia and Empathy in Autism. J. Autism Dev. Disord..

[43] Raimo S., Boccia M., Gaita M., Canino S., Torchia V., Vetere M.A., Di Vita A., Palermo L. (2022). The Bodily Fundament of Empathy: The Role of Action, Nonaction-Oriented and Interoceptive Body Representations. Psychon. Bull. Rev..

[44] Ainley V., Maister L., Tsakiris M. (2015). Heartfelt Empathy? No Association between Interoceptive Awareness, Questionnaire Measures of Empathy, Reading the Mind in the Eyes Task or the Director Task. Front. Psychol..

[45] Shah P., Catmur C., Bird G. (2017). From Heart to Mind: Linking Interoception, Emotion, and Theory of Mind. Cortex.

[46] Schandry R. (1981). Heart Beat Perception and Emotional Experience. Psychophysiology.

[47] Dziobek I., Fleck S., Kalbe E., Rogers K., Hassenstab J., Brand M., Kessler J., Woike J.K., Wolf O.T., Convit A. (2006). Introducing MASC: A Movie for the Assessment of Social Cognition. J. Autism Dev. Disord..

[48] Faul F., Erdfelder E., Buchner A., Lang A.-G. (2009). Statistical Power Analyses Using G*Power 3.1: Tests for Correlation and Regression Analyses. Behav. Res. Methods.

[49] Longarzo M., D’Olimpio F., Chiavazzo A., Santangelo G., Trojano L., Grossi D. (2015). The Relationships between Interoception and Alexithymic Trait. The Self-Awareness Questionnaire in Healthy Subjects. Front. Psychol..

[50] Parsons L.M. (1987). Imagined Spatial Transformations of One’s Hands and Feet. Cogn. Psychol..

[51] Daurat-Hmeljiak C., Stambak M., Berges J. (1978). Il Test dello Schema Corporeo. Una Prova di Conoscenza e Costruzione Dell’immagine del Corpo.

[52] Pino M.C., Pettinelli M., Clementi D., Gianfelice C., Mazza M. (2015). Improvement in cognitive and affective theory of mind with observation and imitation treatment in subjects with schizophrenia. Clin. Neuropsychiatry.

[53] Raimo S., Trojano L., Pappacena S., Alaia R., Spitaleri D., Grossi D., Santangelo G. (2017). Neuropsychological Correlates of Theory of Mind Deficits in Patients with Multiple Sclerosis. Neuropsychology.

[54] Raimo S., Cropano M., Roldán-Tapia M.D., Ammendola L., Malangone D., Santangelo G. (2022). Cognitive and Affective Theory of Mind across Adulthood. Brain Sci..

[55] Blair R.J., Cipolotti L. (2000). Impaired Social Response Reversal: A Case of ‘Acquired Sociopathy’. Brain.

[56] Happé F.G. (1994). An Advanced Test of Theory of Mind: Understanding of Story Characters’ Thoughts and Feelings by Able Autistic, Mentally Handicapped, and Normal Children and Adults. J. Autism Dev. Disord..

[57] Prior M., Marchi S., Sartori G. (2003). Social Cognition and Behavior. A Tool for Assessment.

[58] Iman R.L., Conover W.J. (1979). The Use of the Rank Transform in Regression. Technometrics.

[59] Gabriel E.T., Oberger R., Schmoeger M., Deckert M., Vockh S., Auff E., Willinger U. (2021). Cognitive and Affective Theory of Mind in Adolescence: Developmental Aspects and Associated Neuropsychological Variables. Psychol. Res..

[60] Nejati V., Moradkhani L., Suggate S., Jansen P. (2021). The Impact of Visual-Spatial Abilities on Theory of Mind in Children and Adolescents with Autism Spectrum Disorder. Res. Dev. Disabil..

[61] Adenzato M., Brambilla M., Manenti R., De Lucia L., Trojano L., Garofalo S., Enrici I., Cotelli M. (2017). Gender Differences in Cognitive Theory of Mind Revealed by Transcranial Direct Current Stimulation on Medial Prefrontal Cortex. Sci. Rep..

[62] Russell T.A., Tchanturia K., Rahman Q., Schmidt U. (2007). Sex Differences in Theory of Mind: A Male Advantage on Happé’s “Cartoon” Task. Cogn. Emot..

[63] Spenser K.A., Bull R., Betts L., Winder B. (2022). Gender Differences in Theory of Mind, Empathic Understanding, and Moral Reasoning in an Offending and a Matched Non-Offending Population. Int. J. Offender Ther. Comp. Criminol..

[64] Palser E.R., Palmer C.E., Galvez-Pol A., Hannah R., Fotopoulou A., Kilner J.M. (2018). Alexithymia mediates the relationship between interoceptive sensibility and anxiety. PLoS ONE.

[65] Kano M., Fukudo S. (2013). The Alexithymic Brain: The Neural Pathways Linking Alexithymia to Physical Disorders. BioPsychoSocial Med..

[66] Grynberg D., Chang B., Corneille O., Maurage P., Vermeulen N., Berthoz S., Luminet O. (2012). Alexithymia and the Processing of Emotional Facial Expressions (EFEs): Systematic Review, Unanswered Questions and Further Perspectives. PLoS ONE.

[67] Scarpazza C., di Pellegrino G., Ladavas E. (2014). Emotional Modulation of Touch in Alexithymia. Emotion.

[68] Scarpazza C., Zangrossi A., Huang Y.-C., Sartori G., Massaro S. (2022). Disentangling Interoceptive Abilities in Alexithymia. Psychol. Res..

[69] Garfinkel S.N., Tiley C., O’Keeffe S., Harrison N.A., Seth A.K., Critchley H.D. (2016). Discrepancies between dimensions of interoception in autism: Implications for emotion and anxiety. Biol. Psychol..

[70] Azevedo R.T., Badoud D., Tsakiris M. (2018). Afferent Cardiac Signals Modulate Attentional Engagement to Low Spatial Frequency Fearful Faces. Cortex.

[71] Crespi B., Dinsdale N. (2019). Autism and Psychosis as Diametrical Disorders of Embodiment. Evol. Med. Public Health.

[72] Desmedt O., Heeren A., Corneille O., Luminet O. (2022). What do measures of self-report interoception measure? Insights from a systematic review, latent factor analysis, and network approach. Biol. Psychol.

[73] Crucianelli L., Enmalm A., Ehrsson H.H. (2022). Interoception as independent cardiac, thermosensory, nociceptive, and affective touch perceptual submodalities. Biol. Psychol..

