# Supplementary material for: On the Embodiment of Social Cognition Skills: The Inner and Outer Body Processing Differently Contributes to the Affective and Cognitive Theory of Mind
Source: Brain Sci. 2022 Oct 23;12(11):1423. doi: 10.3390/brainsci12111423 (PMC9688437; doi:10.3390/brainsci12111423)
Supplement: Supplementary file 1 [file brainsci-12-01423-s001.zip › brainsci-1970208-supplementary.pdf]

Supplementary Materials

Table S1. Spearman correlation coefficients between the cognitive and affective components of ToM measures on the one hand and control tasks on the other.

			Object Laterality Task	***Christmas Tree Task***
Cognitive ToM	ATT	r_{rho}	.18	.04
		p	.10	.74
Affective ToM	negEAT	r_{rho}	.19	-.13
		p	.09	.24
	posEAT	r_{rho}	.15	.01
		p	.18	.91

The table shows Spearman correlations between the cognitive (ATT) and affective (negEAT; posEAT) ToM tasks on the one hand and control tasks on the other.
Note: ToM, Theory of Mind; ATT, Advanced Test of ToM; negEAT, negative Emotion Attributions Task; posEAT, positive Emotion Attribution Task.

Table S2. Results of a multiple regression analysis to explore the effect of the FBE and Christmas Tree Task on the Advanced Test of ToM (ATT) performance.

	Predictor	*Beta*	*t*	*p*
**Cognitive ToM**				
ATT	FBE	.30	2.52	.01
	CTT	-.09	-0.77	.44
$R^2 = .08$				
$F(2,79) = 3.23$; $p = .04$				

The table shows the results of the multiple linear regressions performed to explore the role of the nonaction-oriented body representation task (FBE) and the respective control task (CTT) in performing the cognitive ToM task (ATT).
Note: ToM, Theory of Mind; ATT, Advanced Test of ToM; FBE, Frontal-Body Evocation Task; CTT, Christmas Tree Task
